# Pharmacologic challenges in pediatric intestinal failure: A review

**DOI:** 10.1016/j.intf.2025.100039

**Published:** 2025-01-29

**Authors:** Sean Coyne, Kathleen M. Gura

**Affiliations:** a Department of Pharmacy, Boston Children’s Hospital, Boston, MA, USA; b Department of Pharmacy and The Division of Gastroenterology, Hepatology and Nutrition, Boston Children’s Hospital, Boston, MA, USA; c Divison of Nutrition, Harvard Medical School, Boston, MA, USA

**Keywords:** Intestinal failure, Short bowel syndrome, Small bowel bacterial overgrowth, Central line associated bloodstream infection, Antibiotic lock

## Abstract

Although the hallmark of intestinal failure (IF) management — also known as intestinal rehabilitation — is parenteral nutrition, many of the complications that arise from this complex condition require additional pharmacologic interventions to prevent more serious consequences such as intestinal failure associated liver disease and central line associated bloodstream infections. This brief review highlights some of the more commonly used treatment strategies in pediatric patients with IF along with a review how different types of short bowel anatomy impact drug administration.

## Introduction

Providing optimal pharmaceutical care remains a challenge in patients with intestinal failure (IF). For example, the degree of malabsorption will vary after gastrointestinal (GI) resection. In cases where more than 50 % of the small intestine is resected, malabsorption of macro- and micronutrients can occur and may similarly impact the drug absorption process. Complications may arise because of the unique anatomy of these patients or due to the presence of the indwelling devices necessary to deliver the nutrition necessary to sustain them. This review will focus on these challenges and provide an overview of some of the more commonly used medications in this population.

## Impact of resection type on therapeutic response: residual bowel length, location, and absorptive capacity

Resection of the GI tract has significant consequences for the absorption of enterally administered medications. Owing to its large surface area and more permeable membranes in comparison to the stomach, the small intestine is the primary site of drug absorption. Depending on the amount and location of the bowel resected, the number of carriers, channels, or pores used to transport a drug into systemic circulation may be decreased [1]. Similarly, the amount of drug that can be absorbed via active transport across a cell membrane becomes limited. In situations of reduced blood flow (e.g., shock), the concentration gradient of drug across the intestinal mucosa is lowered, which further reduces the amount of drug absorbed by passive diffusion. Treatment failures often occur. Appreciating these differences in patient anatomy when considering drug regimens can often decrease this risk.

Within hours after intestinal resection, the remaining bowel will start to undergo physiological and structural changes. If the jejunum is resected, the ileum will start to adapt and develop the functional and structural characteristics of the jejunum. This is possible because the ileum has superficial crypts and shorter villi. Unfortunately, in the event of an ileal resection, the jejunum is less able to adapt and cannot assume the functions of the ileum. This can further impact drug response. Fortunately, absorptive capacity may improve over time since the date of resection due to bowel adaptation, suggesting time since resection should be taken into account [2].

### Impact of resection on gastric motility

Dysmotility can often occur as a result of resection of the small intestine. Normally, motility in the ileum is three times slower than the jejunum [1]. Depending on the area removed, transit time will vary. Following a jejunal resection, gastric emptying is more rapid; however, intestinal transit time may remain normal due to the ileal brake [3]. In patients with residual bowel in continuity, gastric emptying is slower and similar to an unresected bowel. In contrast, patients with colonic or ileal resection, cells that release peptide YY are missing, resulting in a loss of inhibition of gastric emptying, thus decreasing intestinal transit time [3]. In adults with less than 100 cm of residual jejunum, rapid gastric emptying can decrease drug absorption. Drug concentrations may be altered due to malabsorption coupled with rapid intestinal transit. Delayed gastric emptying can prolong drug release and impact drug absorption.

## Examples of medications impacted by changes in absorption

### Oral contraceptives and steroids

Patients with IF who are receiving oral contraceptives or steroids are at risk for treatment failure, as these agents are primarily absorbed in the small intestine. In patients receiving levonorgestrel, patients with the largest amount of small bowel resected were found to have the lowest serum levels [4]. Moreover, jejunoileal bypass may result in reduced bioavailability of both norethindrone and levonorgestrel [4].

### Cholestyramine

Drug response may be altered depending on the location of the resection because of impaired bile salt reabsorption. For example, if more than 100 cm of ileum is removed, the reabsorption of bile salts into the enterohepatic circulation is impacted, which results in a decrease in the production of bile acid [5]. As more than 95 % of bile salts are recirculated, the liver is unable to synthesize an adequate amount of replacement bile acid. This decreased bile acid pool results in impaired fat digestion and micelle formation. As a consequence, steatorrhea and fat-soluble deficiencies can occur [5]. Thus, in that instance, the use of cholestyramine may actually worsen steatorrhea and should generally be avoided, or at most reserved for those patients with IF with a colon who fail other front-line agents. This is particularly true in individuals with short bowel anatomy type 1 or 2, where the bile acid pool is already reduced [5]. Furthermore, response rates may differ among cholestyramine products due to excipients (e.g., sorbitol, sucrose) which could potentially worsen diarrhea. When treating bile salt-induced diarrhea, the powder form should be used. The area of resection must also be considered when deciding which form of cholestyramine to use. Enteric coated cholestyramine contains cellulose acetate phthalate which releases its active substance in the distal ileum/colon whereas the powder is released only in the small bowel.

In contrast, there are cases where cholestyramine has been found to be useful in managing patients who have bile salt-induced watery diarrhea because as a bile acid sequestrant, it forms a nonabsorbable complex with bile acids in the intestine [6]. Chloride is then released and the enterohepatic reuptake of intestinal bile salts is inhibited [5], [6]. Likewise, patients with short bowel anatomy type 3 (i.e., jejunoileal anastomosis with intact colon), may also benefit from the use of the cholestyramine if diarrhea is caused by the colonic toxicity of poorly absorbed bile salts [6].

### Mesalamine

The type of surgical technique used to perform a resection can also impact drug absorption. For example, depending on the type of anastomosis, patients managed with mesalamine for Crohn’s disease may have different drug concentrations. Mesalamine, when manufactured as a pH-sensitive enteric coated tablet, is designed to release its active drug in the colon. In patients with a small bowel resection, the type of anastomosis (side to side vs. end to end) can influence its effectiveness. In one study patients with end-to-end anastomosis had lower mucosal concentrations of drug and more frequent disease recurrence when compared to those with a side-to-side anastomosis [7]. Side-to-side anastomosis may increase segmental transit time, resulting in improved absorption and higher concentrations of mesalamine in the mucosa.

### Digoxin

Studies investigating digoxin therapy in IF patients who have undergone complete small bowel resection have demonstrated that the colon can compensate and is able to absorb sufficient digoxin to achieve therapeutic levels. In one case report, a patient with only 18 cm of jejunum and without continuity of the small intestine and bowel had severe digoxin malabsorption such that only intravenous administration of the agent was able to achieve adequate serum levels [8]. Conversely, in another patient with an end jejunostomy and only 12–15 cm remaining jejunum, adequate serum levels were achieved despite reduced digoxin absorption when the liquid formulation was used [9].

### Hydrochlorothiazide

Absorption of hydrochlorothiazide, a commonly used thiazide diuretic, has been shown to be impacted by reductions in gastric emptying. In one investigation, patients who had undergone a jejunoileostomy or ileocaecostomy after intestinal shunt surgeries for obesity had a 50 % decrease in hydrochlorothiazide uptake [10]. It was thought that shortened transit time was responsible for the malabsorption experienced [6].

### Antibiotics

The literature is limited regarding the impact of intestinal failure and the absorption of antibiotics. Enteral absorption might be adequate in some SBS patients. Other patient factors besides site and extent of bowel resection (e.g., delayed gastrointestinal transit time) may impact enteral drug absorption. In one study comparing aminopenicillins with other orally administered antibiotics in children with significant small bowel resection of small bowel, Menardi et al. observed similar levels of cephalexin and trimethoprim-sulfamethoxazole between children with SBS and controls, but the concentrations of epicillin (an aminopenicillin) were reduced to only 10 % of therapeutic levels. The authors suggested that oral cephalexin and trimethoprim-sulfa could be used therapeutically in children with short-bowel syndrome. If an aminopenicillin is indicated, parenteral therapy is advised [11]. In adults with IF, Gompelman reported that the bioavailability of enterally administered clindamycin, ciprofloxacin, and fluconazole may be altered. Although clindamycin and fluconazole exhibited > 90 % bioavailability, ciprofloxacin showed markedly decreased bioavailability to only 36 % [12]. These findings suggest that therapeutic drug monitoring is necessary when managing systemic infections with enteral antibiotics in IF patients and that the enteral route may be possible in some patients [2].

## Managing the comorbidities associated with intestinal failure

Patients with IF often require unique therapies to assist in bowel adaptation. Depending on the patient, antimotility, antisecretory and/or prokinetic agents may be required. Due to the complexities of IF, dosage recommendations are often quite different from established norms. For example, in IF gut motor activity is typified by a normal feeding pattern, along with more frequent interdigestive motor complexes (i.e., the periodic pattern of contractions in the stomach and small intestine that occurs between meals) and a marked reduction in phase 2 metabolism (i.e., conjugation) activity, resulting in different responses to medications [13]. Table 1 lists examples of medications commonly used in pediatric IF patients along with relevant dosing information. Figure illustrates the location of sites of drug absorption within the GI tract.

**Table 1:** Examples of medications used in pediatric IF patients.

**Indication**	**Medication**	**Typical dose**	**Comments**
**Anti-motility agents**	**Loperamide**	Usual doses: Infants ≥ 2 months and Children: Oral: 0.08–0.24 mg/kg/day in divided doses every 8–12 h; maximum dose: 2 mg/dose Typical max doses used in IF: 0.8 mg/kg/day or 24 mg/day	Cases of torsades de pointes, cardiac arrest, and death have been reported with the use of a higher than recommended dosage of loperamide. Use tablets, avoid liquid formulations Propylene glycol content may increase output
**Prokinetics**	**Cisapride**	Infants, Children, and Adolescents: Oral: 0.15–0.2 mg/kg/dose 3–4 times/day; maximum dose: 10 mg/dose Note: Doses up to 0.3 mg/kg/dose every 8 h have been described; however, due to safety concerns, most recommend a maximum dose of 0.8 mg/kg/day in divided doses	May cause QT prolongation Numerous drug interactions Only available via compassionate use protocol Administer ≥ 15 min before meals or feeding
	**Domperidone**	Pediatric: 0.25 mg/kg. This should be given up to three times per day with a maximum dose of 0.75 mg/kg per day. Adult: 0 mg 3 times daily (maximum: 30 mg/day). Note: unsuitable for use in adults and adolescents weighing less than 35 kg. Should not be used in children < 12 years of age	Domperidone is available via an Investigational New Drug Application (IND) in the United States for severe GI disorders refractory to standard therapy. For more information on the requirements for the IND, contact the FDA at 301-796-3400.
	**Erythromycin**	Note: Most commonly reported salt form in studies was erythromycin ethylsuccinate (when reported). **Preterm neonates**: PNA ≥ 14 days: Note: Treatment duration is typically 7–14 days. Low-dose regimen: Oral: 1.5–2.5 mg/kg/dose every 6 h or 1 mg/kg/dose every 8 h Intermediate-dose regimen: Oral: 5 mg/kg/dose every 6–8 h. High-dose regimen: Oral: 10–12.5 mg/kg/dose every 6–8 h.; after 2 days, may decrease dose to 4 mg/kg/dose every 6 h for 5 additional days. *Note: High-dose regimens not recommended due to risk of adverse effects and the possibility that therapeutic effects may be reduced* **Infants, Children, and Adolescents:** Response determination during gastric emptying study: IV: 2.8 mg/kg infused over 20 min; maximum dose: 250 mg/dose Treatment: Oral: 3 mg/kg/dose 4 times daily; may increase as needed to effect up to 10 mg/kg/dose; maximum dose: 250 mg/dose	Involved in many drug interactions; prokinetic at low does but causes gastric antral spasm and delayed gastric emptying at high doses; oral route preferred; serious cardiac complications including arrest have occurred with IV administration
	**Azithromycin**	azithromycin use as a prokinetic agent has not widely been researched median dose of azithromycin was 3.97 mg/kg has been used	Risk of antimicrobial resistance Risk of tachyphylaxis, cardiac arrhythmia Azithromycin may be an equal alternative to erythromycin Unlike erythromycin, azithromycin is not extensively metabolized by CYP3A4, may decreases cardiac risk associated with increased blood levels secondary to enzyme inhibition
	**Metoclopramide**	Oral, IM, IV: Neonates, infants and children: 0.4–0.8 mg/kg/d in 4 divided doses **Preterm neonates, PNA ≥ 3 days**: IV: 0.13 mg/kg/dose every 8 h administered 30 min prior to a feed. In a small case series of preterm neonates with persistent feeding intolerance requiring PN; PNA at treatment: 25–70 days), a lower dose of 0.033 mg/kg/dose IV every 8 h was reported effective in all patients **Postpyloric feeding tube placement** Note: Use in patients who have failed conventional measures. Infants and Children < 6 years: IV: 0.1 mg/kg as a single dose. Children ≥ 6 years and Adolescents ≤ 14 years: IV: 2.5–5 mg as a single dose. Adolescents ≥ 15 years: IV: 10 mg as a single dose. **Gastroesophageal reflux, treatment: Note:** Routine use is not recommended; reserve use as a last resort after all other therapies have failed Infants, Children, and Adolescents: Oral: 0.1–0.2 mg/kg/dose every 6–8 h; maximum dose: 10 mg/dose	High incidence of central nervous system effects
**Miscellaneous**	**Octreotide**	Wide dosing variation exists IV, subcutaneous: begin with 1–10 g/kg every 12 h and increase by 0.3 g/kg/dose at 3-d intervals	• Dose titrated to stool output. • May cause hyper- or hypoglycemia. • Based on animal data, suppression of growth hormone may be of concern when used as long-term therapy in children
	**Ursodeoxycholic acid (urso, ursodiol)**	Infants and children: 30 mg/kg/d in 3 divided doses **Parenteral nutrition–induced cholestasis:** Treatment: Preterm and term neonates: Oral: 10–30 mg/kg/day in 3 divided doses Prevention: Preterm neonates: Oral: 20–25 mg/kg/day in 2–4 divided doses Infants and Children: Oral: 30 mg/kg/day in 3 divided doses **Pruritus secondary to cholestasis** Infants, Children, and Adolescents: Oral: 15–20 mg/kg/day once daily or in divided doses twice daily; doses up to 30 mg/kg/day may be necessary in some patients	May cause diarrhea; liquid formulation must be specially compounded.
	**Cholestyramine**	**Diarrhea secondary to intestinal failure, short-bowel syndrome:** Children and Adolescents: Oral: 240 mg/kg/day in 2–3 divided doses; maximum daily dose: 8 g/day has been suggested **Pruritus secondary to cholestasis**: Dosages are expressed in terms of anhydrous resin: Children ≤ 10 years: Oral: 240 mg/kg/day in 2 or 3 divided doses administered in the morning around breakfast and if necessary, the third dose at lunch; may titrate dose to effect. Typical maximum daily dose of 4 g/day; however, higher doses have been reported to treat pruritus patients, higher doses were associated with increased steatorrhea and required dosage reduction. Children > 10 years and Adolescents: Oral: 240 mg/kg/day administered in the morning before breakfast; may titrate dose to effect. Some experts have suggested a maximum daily dose of 8 g/day; in adult patients, the AASLD guidelines recommend an initial dose of 4 g/day; may titrate up to 16 g/day	Interacts with ursodeoxycholic acid. Administer 1 h after ursodiol doses. Therapeutic effect may differ among products due to excipients (e.g., sorbitol, sucrose) which could potentially worsen diarrhea Significant drug interactions exist
	**Teduglutide**	Children and Adolescents weighing ≥ 10 kg: Subcutaneous: 0.05 mg/kg/dose once daily	Reduce dose in renal dysfunction (< 60 mL/min/1.73 m^2^) Rotate injection site between thighs, abdomen, upper arm Development of colorectal polyps has occurred Monitor fluid and electrolyte status REMS drug http://www.gattexrems.com/

## Antidiarrheal agents

Control of diarrhea using an antimotility agent is the mainstay of IF management. Loperamide, an opioid-receptor agonist that inhibits peristalsis, is often used in IF patients to reduce transit rate and enhance absorption [14]. By slowing intestinal motility, water and sodium loss is reduced by approximately 20–30 % from an ileostomy [15]. In recent years, loperamide has become a drug of abuse, with individuals withdrawing from opioids using it to ameliorate their symptoms, or simply to induce euphoria. Loperamide has been available as an over the counter (OTC) medication in the US since 1988. When taken at recommended doses, loperamide does not yield the “high” seen with other opioids. However, at extremely excessive doses (i.e., more than 100–200 mg/day), loperamide can cross the blood-brain barrier and produces effects like those associated with centrally acting opioids like heroin, hydrocodone, or morphine [15]. Loperamide overdoses have been associated with serious cardiac complications, including arrhythmias, loss of consciousness or fainting, and myocardial infarction. In reaction to these concerns, in September 2019, the FDA approved changes to OTC loperamide products. These changes limit each container to no more than 48 mg of loperamide and require the tablets and capsules to be individually packaged (i.e., unit-dosed) [16]. Furthermore, standard doses of loperamide may interact with medications that can cause QT prolongation such as azole antifungal drugs and macrolide antibiotics [15]. This medication should not be used in patients with slow transit times, those with refractory small bowel bacterial overgrowth, or patients with acute GI infections, including *Clostridium difficile*. The liquid formulation should not be used unless the formulation is confirmed to be both sugar free and alcohol-free to avoid the side-effect of osmotic diarrhea [17].

Other opioid derivatives, such as codeine and tincture of opium, should be avoided in children with IF associated diarrhea. Severe adverse side effects have been observed in children receiving codeine, especially if they are CYP2D6 ultra-rapid metabolizers. In such patients, codeine is converted into morphine in the body at a faster rate than normal, resulting in high levels of morphine in the blood that can cause toxic effects including respiratory depression. Because atropine can cross the blood-brain barrier, diphenoxylate/atropine is not recommended as the high doses needed to manage diarrhea has been associated with systemic effects (e.g., sedation) [18].

### Acid reducing agents

Immediately after extensive GI resection, gastrin secretion is increased, resulting in excess gastric acid secretion [19]. Hyperacidic secretions impair carbohydrate and protein digestion, micelle formation, and lipolysis, resulting in malabsorption and diarrhea [20]. Acid blockers, such as histamine-2 receptor antagonists and proton-pump inhibitors, can be used to decrease gastric acid, reduce jejunostomy output, and improve absorption. Parenteral administration may be preferred, especially in those patients with extremely short bowel as the patient’s capacity for enteral absorption may be insufficient to achieve a therapeutic effect. The duration of therapy varies dramatically between clinicians. Given that there is a potential link between acid blockade and an increased risk in infections, including small intestine bacterial overgrowth (SIBO), weaning the patients from this class of medications as soon as possible is preferred [21].

### Prokinetic agents

Intolerance to enteral nutrition is a hallmark of IF with nausea, vomiting, and abdominal distention presenting as common signs and symptoms. Prokinetic agents such as metoclopramide, erythromycin, and cisapride have been shown to promote GI motility [17]. Side effects such as central nervous system effects and cardiotoxicity, however, have limited their usefulness (Table 1)

## Medications targeting gastric emptying

### Octreotide

Octreotide is sometimes used to inhibit intestinal motility and decrease gastric secretions. It does so by slowing of gastric emptying and small bowel transit time and inhibiting the release of peptide hormones. In patients with IF, especially those with high-output end-jejunostomy, octreotide may reduce intestinal efflux, leading to a decrease in volume requirements. Given that octreotide can decrease splanchnic protein synthesis, bowel adaptation can be hindered [22]. Octreotide is primarily administered as a subcutaneous injection. Adverse events to treatment include tachyphylaxis, development of gallstones, and pancreatitis [23]. Data supporting its addition to parenteral nutrition (PN) solutions is conflicting, although the manufacturer states it is incompatible in PN as it can result in the formation of a glycosyl octreotide conjugate thus decreasing its efficacy [24], [25].

### Teduglutide

Gastrointestinal hormones to improve intestinal adaptation have become a recent addition to the various pharmacologic strategies used to manage IF patients. Glucagon-like peptide 2 (GLP-2) is a naturally occurring hormone produced by the enteroendocrine cells in the colon and distal ileum. This hormone delays gastric emptying and induces small intestine epithelial proliferation [26]. Teduglutide, an analogue of GLP-2, is administered as a daily subcutaneous injection and has been found to increase crypt depth and villous height, allowing for improved fluid absorption and a reduced PN requirement. In patients able to fully wean from PN, carefully monitoring is still necessary as there as increased risk of micronutrient and vitamin deficiencies [27].

## Miscellaneous agents

### Ursodeoxycholic acid

Ursodeoxycholic acid (UDCA, ursodiol) has been shown to be an effective choleretic. It decreases intestinal absorption and suppresses hepatic synthesis and storage of cholesterol. This is thought to reduce cholesterol saturation of bile. Although used frequently to manage intestinal failure associated liver disease (IFALD), it may be poorly absorbed in patients without an ileum, thus causing more diarrhea. Lack of enteral feeding may cause gallbladder stasis in these patients and the subsequent reduction in bile salts may cause formation of cholesterol stones [28]. Before the advent of alternative lipid emulsions as an IFALD management strategy, UDCA was frequently used to prevent or treat IFALD [29]. UDCA prevents cytolysis and apoptosis, changes the expression of enzymes and transporters that reduce bile acid cytotoxicity, modulates ductular bile flow, prevents endocytic internalization of canalicular transporters, and has immunomodulatory properties [30]. UDCA has been shown to improve the biochemical and clinical signs and symptoms of IFALD. UDCA may reduce the levels of gamma-glutamyl transferase (GGT), alanine transaminase (ALT), alkaline phosphate (ALP), and direct bilirubin [28], [29], [30].

## Dosage form considerations

**Fig. 1:**
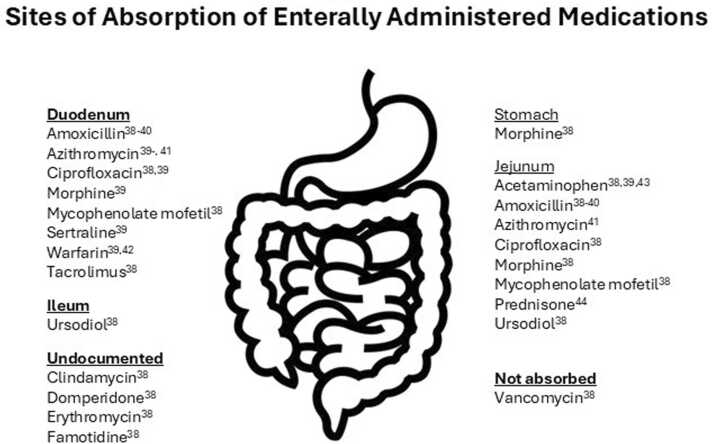
Sites of absorption of enterally administered medications. Ref [32], [33], [34], [35], [36], [37], [38].

In patients in whom significant amounts of small bowel is resected or nonfunctional, managing drug therapy using orally administered medication is a critical but often overlooked component of patient care. As per a report by the American Gastrointestinal Association, “Oral medication absorption is often impaired and larger doses, intravenous, or sublingual (SL) delivery may be required; significant interpatient variability may be observed.” [31]. To further complicate matters, even in a healthy individual without IF, the exact location within the intestinal tract where a medication is absorbed is not known, nor are the specific factors that can impact it, making it even more challenging to determine drug response in a patient with dysmotility or an intestinal resection.(Fig. 1) The function of the remaining bowel, the presence (or absence) of terminal ileum, and the pH within the intestine can all be altered, each factor further influencing the extent of medication absorption. In addition, the type of an orally administered dosage form can also impact drug response in an IF patient. For example, if an IF patient is prescribed a sustained release dosage form, drug response tends to be unreliable due to the variability in drug absorption which is both unpredictable and erratic [31]. To circumvent this, the medication should be converted into the equivalent immediate-release dosage forms.

### Excipients

Medications often contain excipients, inactive substances that are combined with the active ingredient. Excipients may be used to enhance the drug’s therapeutic properties including improving drug absorption, enhancing solubility, improving stability, or improve the smell or taste of an otherwise unpalatable medication. These additives are used in products designed for oral administration; most are simply not designed for administration via a feeding tube. There are significant differences between oral administration and delivery of a medication through a feeding tube. Solid dosage forms such as tablets and capsules have coatings to reduce shelf degradation from moisture and to improve the passage of the medication through the alimentary tract. These coatings reduce surface tension to allow the drug to slide down the throat when administered with water. This type of coating, however, also acts as an adhesive with other tablets when they mixed with an insufficient volume of water, resulting in the tablets to clump [39]. When this occurs when the tablets are given down an enteral feeding tube, clogging can occur. Similarly, this phenomenon can occur when multiple drugs are crushed together for enteral administration.

In addition to obstructing the device, patients using enteral feeding tubes are distinctively sensitive to concentrated medications possessing high osmolarity. Most liquid medication have an osmolarity of more than 1000 mOsm/L [3]. Unfortunately, the tolerable osmolarity of the GI tract is about 285 mOsm/L, thus hypertonic medications require dilution to improve tolerance. This may result in large volumes of water to dilute the dose to a tolerable concentration [3]. Furthermore, the location of the tip of the enteral feeding tube within the GI tract also must be considered. Administration of a liquid medication into the stomach allows the residual fluid volume to help dilute the high osmolarity of the medication, thus improving tolerance. Administration into the lower GI tract (i.e., jejunum) does not afford this protection. The jejunum has no similar residual fluid capacity. The administration of concentrated liquids will cause the bowel to respond by providing adequate dilution of the medication through cramping. This response is more commonly known as osmotic diarrhea [40]. Specific excipients may contribute to intolerance in patients using feeding tubes. Sorbitol is a sweetener that is added to many liquid medications. It is effective at dissolving many water-soluble drugs. The Food and Drug Administration (FDA) recognizes it as General Recognized as Safe (GRAS) group of excipients. Sorbitol, however, is also a potent cathartic. Unfortunately, manufacturers are not required to report the amount of sorbitol present in a medication’s package insert or on the product’s label. To obtain this information, the clinician must contact the manufacturer with the specific lot number. Furthermore, generic products may or may not contain sorbitol, regardless of what the brand name product contains [41]. Moreover, manufacturers can change the amount of sorbitol present in their product without noting it on the label and it can vary with each lot.

## Managing complications specific to IF

### Small bowel bacterial overgrowth (SIBO)

In patients with IF, SIBO is a common finding. Its etiology is multifactorial, with many believing that intestinal dysmotility, altered intestinal anatomy, resection of the ileocecal valve and the use of acid blockers increasing the risk [42]. Moreover, the odds of acquiring a central line associated bloodstream infection (CLABSI) appear to be greater in those with SIBO in comparison to those without, making it imperative that SIBO be prevented. As summarized in Table 2, cyclical rotation (i.e., typically 1 week per month) of broad-spectrum antibiotics (e.g., metronidazole, ciprofloxacin) is often used to manage SIBO symptoms. Ideally, endoscopic sampling and quantitative cultures of duodenal aspirates may be helpful in tailoring therapy [43].

**Table 2:** Comparison of agents used for treatment of bacterial overgrowth, (typical course 7–10 days), all meds given orally!.

Medication	Pediatric dose	Adult dose	Comments	% orally Absorbed	% renally excreted
Amphotericin B Deoxycholate	< 5 years: 100 mg bid 5–12 years: 250 mg bid	500 mg bid	Injection given orally	9	40 % (2–5 % active)
Augmentin (Amoxicillin/ clavulanic acid)	10 mg/kg/dose bid	500 mg bid		Complete (amoxicillin)	30–40 %
Bactrim (TMP/SMX)	2 mg TMP/kg/dose daily	1 SS tablet daily Each tablet: Sulfamethoxazole 400 mg/trimethoprim 80 mg		Almost completely, 90–100 %	Sulfamethoxazole, 10–30 %; Trimethoprim, 50–75 %
Ciprofloxacin	20–40 mg/kg/day bid	500 mg bid		50–80 %	30–50 %
Clindamycin	10–30 mg/kg/day Divided tid/qid	300 mg tid		90 %	10 %
Colistin	< 5 years: 25 mg 2–4 times/day 5–12 years: 50 mg 2–4 times/day	100 mg 2–4 times/day	Injection given orally	insignificant	75 % in 24 h
Doxycycline	children > than 8 yrs 100 mg bid	100 mg bid		100 %	23 %
Gentamicin	2 mg/kg/dose bid Others: 2.5 mg/kg/dose tid not to exceed 300 mg/day	2–2.5 mg/kg/dose tid Not to exceed 300 mg	Injection given orally	None	100 %
Metronidazole	10 mg/kg/dose bid others: 5–10 mg/kg/dose bid – tid	250–500 mg tid-qid		90 %	10 %
Neomycin	50 mg/kg/day Divided every 6 hours	500 mg bid 500 mg -2 g every 6–8 h	Available as tablets only	3 %	0.9–1.5 %
Tetracycline	Children > 8 years: 25–50 mg/kg/day in divided doses every 6 hours	500 mg tid		75 %	60 %
Tobramycin	< 5 years: 10 mg 2–4 times/day 5–12 years: 40 mg 2–4 times/day	80 mg 2–4 times/day	Injection given orally	Poor	90–95 %
Rifaximin	Not established 20–30 mg/kg/day has been used	400 mg tid		< 0.4 %	< 1 %
Vancomycin	125 mg every 6 h (10 mg/kg/dose qid) Max total daily dose 2 g/day	125 mg - 500 mg every 6 h Max total daily dose 2 g/day		Poor	Oral doses primarily via feces

### CLABSI prevention and treatment

Due to their reliance on PN and the need for a central venous catheter (CVC) to deliver it, CLABSIs are a risk for patients with IF. In the IF patient, SIBO leading to intestinal translocation and skin contamination can result in different organisms causing CLABSIs. In addition to good hand hygiene and strict aseptic technique when managing the CVC, locking solutions are often used. Concentrated antibiotic solutions are instilled into the CVC lumen and allowed to dwell for a defined period (at minimum 2 h) with the goal of maintaining drug concentrations within the CVC that are high enough to penetrate the biofilm and kill the bacteria within [44]. When treating a CALBSI, systemic antibiotics are used along with the antibiotic lock. Ethanol has been used as an alternative locking agent as it possesses both bactericidal and fungicidal properties. Unfortunately, ethanol is not compatible with all types of CVC materials and may increase the risk for catheter occlusion and breakage [44]. Ethanol is also not compatible with heparin and, if injected through the CVC rather than aspirated, may cause a disulfiram-like reaction in patients receiving metronidazole. Table 3 summarizes commonly used locking solutions.

**Table 3:** Catheter lock solutions.

**Agent**	**Mechanism of action**	**Comments**
**Heparin**	binds to antithrombin and inactivates thrombin and other coagulation factors. This prevents the formation of clots and prolongs blood clotting time Does not have fibrinolytic activity; it will not lyse existing clots	Considered the gold standard May not be any better than NS lock [45] Only product with an FDA labeled indication for VAD locking As much as 20 % may leak into system circulation due to parabolic flow within the catheter Can predispose patient to HIT (heparin induced thrombocytopenia) If dosed too low, increase risk of thrombosis. May induce biofilm formation in presence of Staph aureus Higher heparin concentrations also increase rate of biofilm formation
**Ethanol Lock**	**NO ANTICOAGULANT ACTIVITY** Nonspecific protein denaturation Can penetrate and sterilize biofilms	Not FDA approved indication (off label use of an FDA approved product) Demonstrated efficacy for preventing/treating CLABSI Typically, 70 % concentration used – requires compounding Frequency and dwell times vary (Daily, weekly; 15 min- 24 h) ***Not compatible with all CVC materials*** Polyurethane changes when exposed to ethanol - Increased wall thickness- Reduced elasticity- Elution of catheter materials- Increases risk of catheter rupture /splitting **Compatible with silicone**
**Citrate Locks**	prevents activation of calcium-dependent coagulation pathways (chelates calcium)	4 % citrate at least as effective as heparin Antimicrobial properties at high concentrations Adverse effects Metallic taste, perioral or peripheral paresthesia, one case of fatal cardiac arrhythmia High concentrations can lead to protein precipitation in HD catheters leading to PE Problem in US market: only available in 250 mL or 500 mL bags (used in plasmapheresis)
**Sodium Bicarbonate Locks**	has both anti-infective and anticoagulation properties	Fairly safe Alternative to citrate containing tricitrasol or heparin locks Inexpensive Initially used in hemodialysis catheters, used at US sites without access to ethanol 7.5–8.4 % NaHCO3 used = pH 7–8.5
**4 % Tetrasodium Ethylene-diaminete-traacetic Acid (EDTA)**	Improves catheter patency by working as an in vitro anticoagulant with in vitro antimicrobial activity Active against biofilm-forming Gram positive and negative bacteria, fungi and yeast EDTA – anticoagulant	Dilution of EDTA in sterile water for injection Approved by Health Canada in 2016 as a Class II Medical Device Also approved for use in Europe and Australia Not approved in US Available through compassionate use protocols
**Taurolidine and Heparin**	Heparin binds to antithrombin and inactivates thrombin and other coagulation factors; does not have fibrinolytic activity; will not lyse existing clots Taurolidine is an antimicrobial; its methylol groups bind irreversibly to the cell walls of bacteria, fungi, and some viruses, causing cell death. Also has anti-inflammatory properties	Currently approved only for patients with kidney failure receiving chronic hemodialysis (HD) through a central venous catheter Taurolidine 13.5 mg/mL and heparin 1000 USP Units/mL Taurolidine/heparin is **not** intended for systemic administration May cause HIT

Refs. [45], [46], [47], [48].

## Conclusion

The full spectrum of IF continues to evolve as new therapies emerge to prevent significant complications such as SIBO and CLABSI. The management of IF is multidisciplinary and should be individualized for each patient. This may consist of optimizing nutritional strategy, using pharmacologic and microbiome therapies, preventing CLABSI and sepsis, and minimizing avoidable complications such as diarrhea due to drug excipients. Additional research is needed in order to develop more targeted metabolomics approach and possible treatment options based on the pathogenesis of these comorbidities. Moreover, the incorporation of a clinical pharmacist as a member of the interdisciplinary team is essential for management of the IF patient as they can optimize treatment regimens and minimize adverse effects in these very complex and unique patients.

## Ethical Statement

This manuscript did not require IRB approval because as it was a review paper summarizing highlights of a roundtable session held during the 2024 Pediatric Intestinal Failure Symposium in Pittsburgh, PA in September 2024.

## Patient's/Guardian's consent

Not applicable.

## Funding Statement

The author did not receive any internal or external funding for the preparation of this manuscript.

## CRediT authorship contribution statement

**Coyne Sean:** Writing – review & editing. **Gura Kathleen M:** Writing – review & editing, Writing – original draft, Project administration, Conceptualization.

## Declaration of Competing Interest

The authors declare the following financial interests/personal relationships which may be considered as potential competing interests: Kathleen M Gura reports a relationship with SterileCare that includes: funding grants. Author received research support in form of donated drug from SterileCare as part of a compassionate use protocol. If there are other authors, they declare that they have no known competing financial interests or personal relationships that could have appeared to influence the work reported in this paper.
